# Supplementary material for: Tracking prototype and exemplar representations in the brain across learning
Source: eLife. 2020 Nov 26;9:e59360. doi: 10.7554/eLife.59360 (PMC7746231; doi:10.7554/eLife.59360)
Supplement: Transparent reporting form [file elife-59360-transrepform.docx]

***eLife’s* transparent reporting form**

We encourage authors to provide detailed information *within their submission* to facilitate the interpretation and replication of experiments. Authors can upload supporting documentation to indicate the use of appropriate reporting guidelines for health-related research (see [EQUATOR Network](http://www.equator-network.org/%20)), life science research (see the [BioSharing Information Resource](https://biosharing.org/)), or the [ARRIVE guidelines](http://www.plosbiology.org/article/info:doi/10.1371/journal.pbio.1000412) for reporting work involving animal research. Where applicable, authors should refer to any relevant reporting standards documents in this form.

If you have any questions, please consult our Journal Policies and/or contact us: [editorial@elifesciences.org](mailto:editorial@elifesciences.org).

**Sample-size estimation**

- You should state whether an appropriate sample size was computed when the study was being designed
- You should state the statistical method of sample size computation and any required assumptions
- If no explicit power analysis was used, you should describe how you decided what sample (replicate) size (number) to use

Please outline where this information can be found within the submission (e.g., sections or figure legends), or explain why this information doesn’t apply to your submission:

This information is provided as part of the Participants section of the Methods on page 25:

Forty volunteers were recruited from the University of Oregon and surrounding community and were financially compensated for their research participation. This sample size was determined based on effect sizes for neural prototype-tracking and exemplar-tracking regions estimated from prior studies (Bowman & Zeithamova, 2018; Mack et al., 2013), allowing for detection of the minimum effect size (prototype correlates in anterior hippocampus, d = 0.43 with n = 29) using a one-tailed, one-sample t-test with at least 80% power.

**Replicates**

- You should report how often each experiment was performed
- You should include a definition of biological versus technical replication
- The data obtained should be provided and sufficient information should be provided to indicate the number of independent biological and/or technical replicates
- If you encountered any outliers, you should describe how these were handled
- Criteria for exclusion/inclusion of data should be clearly stated
- High-throughput sequence data should be uploaded before submission, with a private link for reviewers provided (these are available from both GEO and ArrayExpress)

Please outline where this information can be found within the submission (e.g., sections or figure legends), or explain why this information doesn’t apply to your submission:

Information on inclusion/exclusion of data is provided as part of the Participants section of the Methods on pages 25-26:

A total of 11 subjects were excluded. Six subjects were excluded prior to fMRI analyses: 3 subjects for chance performance (< .6 by the end of the training phase and/or < .6 for trained items in the final test), 1 subject for excessive motion (> 1.5 mm within multiple runs), and 2 subjects for failure to complete all phases. An additional 5 subjects were excluded for high correlation between fMRI regressors that precluded model-based fMRI analyses of the first or second half of learning phase: 3 subjects had r > .9 for prototype and exemplar regressors and 2 subjects had a rank deficient design matrix driven by a lack of trial-by-trial variability in the exemplar predictor. In all 5 participants, attentional weight parameter estimates from both models indicated that most stimulus dimensions were ignored, which in some cases may lead to a lack of variability in model fits. This left 29 subjects (age: *M* = 21.9 years, *SD* = 3.3 years, range 18-30 years; 19 females) reported in all analyses. Additionally, we excluded single runs from three subjects who had excessive motion limited to that single run.

**Statistical reporting**

- Statistical analysis methods should be described and justified
- Raw data should be presented in figures whenever informative to do so (typically when N per group is less than 10)
- For each experiment, you should identify the statistical tests used, exact values of N, definitions of center, methods of multiple test correction, and dispersion and precision measures (e.g., mean, median, SD, SEM, confidence intervals; and, for the major substantive results, a measure of effect size (e.g., Pearson's r, Cohen's d)
- Report exact p-values wherever possible alongside the summary statistics and 95% confidence intervals. These should be reported for all key questions and not only when the p-value is less than 0.05.

Please outline where this information can be found within the submission (e.g., sections or figure legends), or explain why this information doesn’t apply to your submission:

We included a Statistical Analyses section as part of the Methods to describe all statistical testing procedures for behavioral and fMRI data (pages 29-37). Results include means, standard deviations, confidence intervals, and effect sizes. Exact p-values are reported to up three decimal places (pages 10-18).

(For large datasets, or papers with a very large number of statistical tests, you may upload a single table file with tests, Ns, etc., with reference to sections in the manuscript.)

**Group allocation**

- Indicate how samples were allocated into experimental groups (in the case of clinical studies, please specify allocation to treatment method); if randomization was used, please also state if restricted randomization was applied
- Indicate if masking was used during group allocation, data collection and/or data analysis

Please outline where this information can be found within the submission (e.g., sections or figure legends), or explain why this information doesn’t apply to your submission:

All experimental manipulations were within-subject, so group assignment is not relevant to the current submission.

**Additional data files (“source data”)**

- We encourage you to upload relevant additional data files, such as numerical data that are represented as a graph in a figure, or as a summary table
- Where provided, these should be in the most useful format, and they can be uploaded as “Source data” files linked to a main figure or table
- Include model definition files including the full list of parameters used
- Include code used for data analysis (e.g., R, MatLab)
- Avoid stating that data files are “available upon request”

Please indicate the figures or tables for which source data files have been provided:

All source data for behavioral, modeling, and model-based MRI analyses (Figures 3-5) are included. Raw fMRI have been uploaded to openneuro.org and will be publically available upon publication of the manuscript: <openneuro.org/datasets/ds002813>
